# Evaluating Artificial Intelligence on the Efficacy of Preference Assessments for Preservice Speech-Language Pathologists

**DOI:** 10.1007/s10882-024-09976-2

**Published:** 2024-07-02

**Authors:** Brenna Griffen, Elizabeth R. Lorah, Christine Holyfield, Nicolette Caldwell, John Nosek

**Affiliations:** 1 Louisiana State University- Shreveport, Department of Psychology, Shreveport, LA USA; 2 University of Arkansas, Department of Curriculum and Instruction, Fayetteville, AR USA; 3 University of Arkansas, Department of Communication Sciences and Disorders, Fayetteville, AR USA; 4 Temple University, Department of Computer and Information Sciences, Philadelphia, PA USA

**Keywords:** Preference assessment, Artificial intelligence, Preservice training, Speech-language pathologists

## Abstract

Individuals with intellectual and developmental disabilities (IDD) face many barriers to meaningful inclusion, including limited language and communication skills. Professionals, such as speech-language pathologists (SLPs), can provide personalized instruction to promote skill development and inclusion. Providing opportunities for individuals to express preferences and choice, such as the multiple stimulus without replacement preference assessment (MSWO; DeLeon & Iwata 1996), within these programs, further increases skill acquisition and social interaction. However, limitations in professionals’ knowledge and skills in performing assessments can be another barrier to meaningful inclusion for individuals with IDD and traditional training methods can be challenging and time consuming. The purpose of the current study was to compare the use of artificial intelligence with traditional pen and paper self-instructional MSWO training methods for five preservice SLPs. Fidelity of implementation and duration of assessment were measured. Results demonstrated a large increase in implementation fidelity for two participants, a moderate increase for two participants and a slight increase for the remaining participant while using artificial intelligence. All participants demonstrated a decrease in scoring errors using artificial intelligence. Regarding duration of implementation, artificial intelligence resulted in a significant reduction for four participants and a moderate reduction for the remaining participant. Results of the follow-up survey suggest that all adult participants and both child participants found that artificial intelligence had a higher treatment acceptability and was more effective at producing socially significant outcomes than traditional methods. Recommendations for clinicians and future research are discussed.

One of the guiding principles for professionals working with individuals with intellectual and developmental disabilities (IDD) is that they have a right to be given opportunities for full and effective participation and inclusion in society and their local communities (Overmars-Marx et al., 2014). Although access to community-based settings has increased for individuals with IDD over the last few decades, many barriers still exist for meaningful inclusion and engagement in basic life activities, such as work, education, health care, and social interactions (Cobigo et al., 2012; Cummins & Lau, 2003). One of the most cited barriers to meaningful social inclusion is the individual with IDD’s lack of necessary knowledge and skills in areas such as literacy, numeracy, communication, and conventional social interaction (Abbott & McConkey, 2006; Overmars-Marx et al., 2014). Oftentimes, a variety of professionals, such as speech-language pathologists (SLPs), are needed to provide personalized direct instruction with specialized interventions to support the development of these skills and facilitate success in community-based settings (Overmars-Marx et al., 2014; Thorn et al., 2009).

SLPs play a vital role by teaching essential communication and literacy skills related to back-and-forth conversations, joint attention, linguistic and prelinguistic communication, peer play, and responses to invitations and greetings, which are foundational for employment, friendship, and meaningful interactions within communities (Wilkinson, 2017). By incorporating opportunities for individuals with IDD to express their preferences within these programs and procedures, SLPs and other professionals can support client control, promote choice-making, and foster autonomy (Rajaraman et al., 2022). Instructional programs that incorporate preferred items have been shown to increase engagement and enjoyment, as well as enhance skill acquisition and promote prosocial behavior (Hu et al., 2021; Logan & Gast, 2001; Phillips et al., 2017). Developmental programming in many community –based settings, such as group homes, public schools and residential schools regularly integrate preferred community-based activities to promote inclusion and responding across environments (Almeida et al., 2018). One of the most commonly used instructional strategies in such programs is positive reinforcement, which is a consequent strategy that involves presenting the learner with a preferred item or activity contingent on specific behaviors leading to increases in the behavior in the future (Cooper et al., 2020; Steinbrenner et al., 2020). Labeled as an evidence-based practice by the National Clearinghouse on Autism Evidence and Practice, research has consistently demonstrated that reinforcement improves outcomes across a wide range of developmental domains, including social interaction, communication, and functional living skills which are vital to community engagement and participation (Gillon et al., 2017; Steinbrenner et al., 2020).

Research has demonstrated a strong correlation between the relative preference for a stimulus and its efficacy as a reinforcer (Call et al., 2012). Therefore, the accurate identification of preferred stimuli is often a first and crucial step in instructional programs (Bishop & Kenzer, 2012). However, this can be especially challenging for individuals with IDD who may be unable to clearly articulate their preferences or answer questions, such as “What do you like?” due to limited communication skills (Brodhead et al., 2016; Chappell et al., 2009). This often leads to professionals relying on caregiver or teacher reports about preferences, but research has shown these reports are often unreliable in identifying effective reinforcers (Brodhead et al., 2017; Cannella-Malone et al., 2005, 2013; Graff & Karsten, 2012a).

Formal stimulus preference assessments (SPA) provide a more accurate measure of preferred stimuli by analyzing a pattern of choosing when the same options are offered consistently over time (Bishop & Kenzer, 2012; Graff & Karsten, 2012a; Logan & Gast, 2001; Virues-Ortega et al., 2014). For example, the Multiple Stimulus Without Replacement (MSWO; DeLeon & Iwata, 1996) is a systematic manipulation of stimuli that can generate a hierarchy of preferred stimuli by calculating the percentage of approach responses for each stimulus (Graff & Karsten, 2012a). Preference hierarchies are unique to each individual and change regularly as preferences shift (Chappell et al., 2009). Professionals can use these hierarchies during instructional sessions to easily transition between preferred items if one item loses its reinforcing efficacy due to satiation effects or shifting motivation (Chappell et al., 2009; Kang et al., 2013).

Despite the benefits of formal SPA, many professionals do not conduct them regularly. A survey of professionals working with children with developmental disabilities identified lack of time as the number one barrier to regular implementation (Graff & Karsten, 2012a). Lack of procedural knowledge was identified as the second largest barrier (Graff & Karsten, 2012a). Given the importance of understanding client preferences, a lack of knowledge and skills in conducting preference assessment procedures on the part of professionals can serve as barriers to skill acquisition and social interaction for individuals with IDD. When these barriers are examined together, it is clear that time-saving methods for training and implementation of SPA procedures would be of great value to professionals.

Artificial intelligence (AI) systems may provide possible solutions to reduce limitations in SPA training and implementation. Although experts have yet to agree on a definition of AI, these systems generally employ a machine capable of imitating intelligent human behavior and are used in tasks that involve complex human actions (Hopcan et al., 2023). AI technologies have many applications across a wide range of fields, including medical, science, art, criminal justice, and advertising (Kelly et al., 2023; Sadiku et al., 2021). Most people interact with AI systems daily in “smart” technologies, such as phones, refrigerators, cars, and watches (Kelly et al., 2023). AI can be divided into three categories: Artificial General Intelligence (AGI), Artificial Narrow Intelligence (ANI), and Artificial Super Intelligence (ASI). Both AGI and ASI are currently in the theoretical stage of development. When fully developed, AGI will be able to achieve goals autonomously and transfer knowledge across scenarios. ASI will be able to function on a higher level of intelligence than humans and will be able to pioneer discoveries in scientific, academic, creative, and social fields. These systems stand in contrast to ANI systems, which rely on machine learning (ML) and natural language processing (NLP) for functionality and are limited in their ability to transfer knowledge between tasks. ANI systems include Apple Siri and Amazon Alexa, which use both ML and NLP for voice recognition software (Kelly et al., 2023).

ANI systems come in many different forms from social media platforms (e.g. Instagram, LinkedIn, and Facebook) to search engines (e.g. Google, Bing, and Yahoo!) to chatbots provided on customer service websites. These systems use ML to find patterns, draw conclusions, and perform tasks without specific instructions or explicit programming through the use of algorithms and statistical models. ML allows AI to respond to in-the-moment changes that occur in the environment. However, this information is only useful when it can be conveyed in human language. This is where NLP comes in to essentially translate computer language into human language (Sadiku et al., 2021). There are many computer programs and tablet-based applications that use AI to support individuals with complex needs. AI can provide personalizing learning, improve the effectiveness of learning environments, increase active engagement in learning, and provide step by step guidance to instructors during instructional programs (Drigas & Ioannidou, 2012; Griffen et al., 2023). Research supports the use of AI to teach children with autism spectrum disorder a diverse range of skills, including communication, social interaction, literacy, academic, daily living skills, and fine motor skills (Hopcan et al., 2023). An advantage of AI is its ability to monitor students’ acquisition of skills in real-time and provide immediate feedback on student progress (Hopcan et al., 2023).

One AI system that provides this type of support is a tablet-based application called Guidance, Assessment, and Information System (GAINS^®^). The main purpose of GAINS is to provide real-time assistance to professionals working with children and coach them through complex, evidence-based instructional programs (Griffen et al., 2023). A unique feature of GAINS is that it actively responds to user input and coaches the user to decide which, if any, action is appropriate based on the data the user provides. Although the program must be set up beforehand by experts, it can be used indefinitely by any number of professionals without the physical presence of an expert. In addition, because GAINS provides training while the professional is with the learner, it redistributes work and time resources away from independent training and to interaction with the learner (Griffen et al., 2023). Instructions are provided visually on the screen of the tablet or smart phone and provided through audio with Bluetooth headsets. GAINS also creates graphical reports that illustrate the results of assessments and interventions, allowing professionals to make data-based treatment decisions more efficiently. Another main advantage is that since guidance remains consistently available on the device, it creates a digital job aide that allows implementation fidelity to remain at high levels over extended time periods. Preliminary research has demonstrated that GAINS can be used to increase implementer fidelity during least-to-most prompting, time delay, and total task chaining procedures for behavior technicians working with children with IDD (Griffen et al., 2023).

Interventions must continue to evolve to better support access to community inclusion for individuals with IDD. This includes interventions to build language and communication that is required for full community participation. AI technology has the potential to support the advancement of such interventions. Given the importance of conducting SPA, such as the MSWO, to identify preferences for individuals with IDD for effectively supporting communication, and the barriers to training and implementation faced by professionals, the purpose of this study was to compare the use of AI (the GAINS application) to typical pen and paper self-instructional methods during preservice SLPs’ training and implementation of MSWO in terms of both fidelity of implementation and duration of assessment. The authors chose traditional pen and paper self-instructional methods as a comparison condition because these methods are commonly used in schools and clinics and recommended by many professionals (Chazin & Ledford, 2016). Additionally, previous research has used pen and paper methods as a standard of comparison to other types of preference assessment training (Hansard & Kazemi, 2018; Higgins et al., 2017; Lim & Hu, 2020; Lipschultz et al., 2015; Nottingham et al., 2018; Rosales et al., 2015; Roscoe & Fisher, 2008). A secondary purpose of this study was to examine adult and child participants’ preference for and perceptions of the treatment acceptability of both methods.

## Methods

### Participants

#### Adult Participants

 Demographic information for adult participants was collected through a written survey prior to beginning the study. All five adult participants were females between the ages of twenty and twenty-two. All participants had less than a year of experience working in the field, no previous training with SPA, and had never conducted SPA. Jill, Molly, and Shawna identified as White/Non-Hispanic. Yazmina identified as White and Hispanic or Latino. Adrianna identified as Hispanic. Jill, Yazmina, and Adriana were juniors. Molly was a sophomore and Shawna was a first-year graduate student. Participants were recruited through flyers posted around the university campus and emails sent to Communication Sciences and Disorders students from the university. Inclusion criteria were that each participant was a student at the university in the Communication Sciences and Disorders department and had no history of conducting or training in MSWO procedures regardless of experience working with children.

#### Simulated Learner

The simulated learner was an adult who performed the role of a child learner participating in the MSWO for all sessions, except generalization probes. The primary interventionist, who was a doctoral student in the field of special education played the role of the simulated learner for all participants across conditions. The simulated learner did not provide any feedback on procedures and performed the actions as outlined in prewritten scripts (adapted from Kuhn, 2017). The simulated learner and scripts were used to control for confederate behaviors that might vary across participants. The scripts included instructions on making selections and how long to pause before making a selection. The scripts also included instances of selecting multiple items and making no selection. One of the three different scripts were randomly chosen prior to beginning the session for both conditions and the adult participants were blind to which script the simulated learner was using. The same script was never used for two sessions in a row with any participant. The simulated learner’s adherence to the script was evaluated as a part of the procedural fidelity measure.

#### Child Participants

The child participants were two neuro-typically developing White/Non-Hispanic or Latino males, ages four and five, recruited from the community. Demographic information was collected for the children, including participant age and ethnicity prior to beginning the study. They only participated in generalization probes at the beginning and end of the study.

#### Informed Consent

Informed consent was obtained prior to beginning the study by the primary interventionist meeting face-to-face with each adult participant and with the parent/guardian of each child participant prior to beginning the study. Following the meeting, each adult signed a written consent form.

### Settings and Materials

This study took place in a room at the university’s clinic that contained a wide variety of children’s toys and activities. Items for the simulated learner were chosen at random and sessions were conducted at an adult-sized table. During the generalization probes, a free operant (FO) preference assessment was conducted by the primary interventionist prior to beginning with the child participants to identify seven to eight stimuli that they interacted with (or consumed) consistently to use during the MSWO. Each generalization session was conducted at a child-sized table. The study used a brief MSWO with each session consisting of three trials, or presentations of the full array of stimuli.

During pen and paper self-instructional condition, written instructions of procedures adapted from DeLeon and Iwata (1996) and used in Higgins et al. (2017) were given to participants (see Appendix A). The use of written instructions instead of other training techniques was chosen based on its use in previous studies as a baseline or comparison condition (Hansard & Kazemi, 2018; Higgins et al., 2017; Rosales et al., 2015; Roscoe & Fisher, 2008; Roscoe et al., 2006). In addition, the participant was provided with a paper data sheet (adapted from Higgins et al., 2017) to collect data on the learner’s responses and help with scoring the MSWO.

During the AI condition, the GAINS application was accessed via tablet-based devices. Participants were able to access audio instructions through AfterShokz^®^ OpenMove^™^ Wireless Bluetooth headsets. Videos of sessions were recorded via a separate tablet-based device and used to help facilitate data collection.

### Interventionists and Training

The primary interventionist was a doctoral student and Board Certified Behavior Analyst (BCBA) with twelve years of experience working with children with IDD. The primary interventionist was trained in performing all types of SPA during coursework, practicum experiences, and clinical training using both didactic training and behavioral skills training from other professionals. An additional graduate student in the Communication Disorders department was used as a secondary observer to collect interobserver agreement (IOA). The primary interventionist reviewed data collection methods with the secondary observer and the secondary observer practiced scoring sample videos until proficient in data collection methods.

### Dependent Measures

The primary dependent measure was fidelity of implementation of MSWO procedures, as assessed with the Fidelity Checklist provided in Appendix B (adapted from Pellegrino, 2019). In this checklist, MSWO procedures were divided into twenty-three steps. Due to the complex nature of the procedure, each step was further broken down into discrete components, for a total of one hundred and thirty-seven components across three trials. An independent observer marked a “ + ” for each component performed independently and correctly, a “–” for each component performed incorrectly, and a “N/A” if the component did not apply. An independent and correct response was defined as completing the component as defined on the Fidelity Checklist. For example, step five (item selection) contains three components. The first component is “the learner is allowed up to 10 s for selection.” If the learner selected an item before the 10 s or the participant removed the items after 10 s, the observer scored a “ + ” for that component. If the participant removed the items before 10 s passed, the observer scored a “-” for the component. The second component of step five (item selection) is “the learner is blocked from accessing items if more than one item is selected.” If the learner did not attempt to select multiple items, the observer marked “N/A.” However, if the learner attempted to select multiple items and access was blocked, the observer marked “ + ”. If the learner attempted to select multiple items and access was not blocked, the observer marked “-”. After each trial, the number of components marked with “ + ” and “N/A” was counted. One session consisted of three trials. Following the third trial, the total number of components marked with a “ + ” or “N/A” from all three trials was added together and divided by the total number of components (one hundred and thirty-seven) and then multiplied by 100 to obtain a percentage correct implementation for the entire session.

The secondary dependent measure was the duration of the assessment. A timer was started as soon as the child participant or simulated learner was seated at the table. The timer was stopped after the participant completed the MSWO and calculated the results, generating a preference hierarchy. Videos of sessions were recorded by the primary interventionist and used to assist with data collection and IOA.

Social validity for adult participants was measured using a System Usability Scale survey (Bangor et al., 2008) administered to each adult participant upon completion of the study (see Appendix C). The survey was divided into three sections: Experiences with GAINS, features of GAINS, and using GAINS for preference assessment. It contained a total of 33 statements and used a Likert-type scale of responding, in which the respondent marked “strongly disagree,” “disagree,” “neutral,” “agree,” and “strongly agree” for each statement.

Social validity for the child participants was measured by a questionnaire given orally to the child participants after each day of the study (see Appendix D). The questionnaire consisted of five questions related to the treatment acceptability of using pen and paper, the treatment acceptability of using AI, conducting SPA, and participating in the study. The child participants were also shown a visual of a green smiley face, yellow face with a straight mouth, and red frowning face and allowed to respond to the questions by pointing at the faces instead of or in addition to responding verbally.

### Interobserver Agreement and Procedural Fidelity

IOA data were collected for 52% (range 40–60%) of sessions in the AI condition, 60% (range 40–80%) of sessions in the pen and paper condition, and 50% of sessions during generalization across participants by watching the video recordings. IOA data for each participant and each phase is displayed in Table 1. IOA for the primary measure of fidelity of implementation were calculated by dividing the number of agreements on the Fidelity Checklist by the number of agreements plus disagreements and multiplying by 100 to obtain a percentage of agreement.

**Table 1:** Interobserver agreement data

Participant Name	AI Condition Average	AI Condition Range	Pen and Paper Condition Average	Pen and Paper Condition Range	Generalization AI Condition	Generalization Pen and Paper
Jill	95.38%	91.24–98.54%	91.24%	90.51–91.97%	89.05%	84.67%
Yazmina	95.38%	94.89–95.62%	92.94%	88.32–95.62%	96.34%	93.43%
Molly	94.65%	89.78–97.81%	93.25%	84.67–96.35%	100%	84.67%
Shawna	92.34%	88.32–96.35%	94.89%	92.70–98.54%	95.62%	91.97%
Adriana	86.86%	85.40–88.32%	89.05%	83.94–94.16%	83.94%	89.78%

IOA for duration were counted as correct if the total duration counted by each observer was within 10 s of each other. IOA for duration was 100% across all conditions for all participants. Since the video recorded each session, any discrepancies were resolved by referring back to the timer on the video.

Procedural fidelity consisted of a five-item questionnaire that was completed by the primary interventionist following every session. The questionnaire concerned the primary interventionist’s implementation of the intervention without offering feedback or guidance before, during or after the MSWO and the simulated learner’s adherence to the written script. IOA on procedural fidelity were collected by the secondary observer for 52% of sessions in the AI condition, 60% of sessions in the pen and paper condition, and 50% of sessions during generalization across participants by watching the video recordings. IOA were calculated by taking the number of agreements on the questionnaire divided by the number of agreements plus disagreements (five total) and multiplied by 100 to obtain a percentage of agreement.

Procedural fidelity data were documented for 100% of sessions across all participants and averaged 100% across all participants for all conditions. IOA for procedural fidelity were collected in all the sessions where IOA on primary and secondary measures were collected and was 100% for all participants for all conditions.

### Experimental Design

This study used a single-subject alternating treatment design (Ledford & Gast, 2018), consisting of two conditions: 1) pen and paper, 2) the AI application (GAINS). Each condition consisted of five sessions for each participant. Prior to beginning the study, each adult participant was randomly assigned to begin in either the pen and paper or AI condition. Afterward, sessions were alternated between the two conditions with two to four days between each condition to control for rapid alternation effects. In addition, generalization probes with children were conducted at the beginning and the end of the study. After five sessions of each condition, the final generalization probe occurred. At this point, the study was concluded.

### Procedures

#### MSWO Procedures

MSWO procedures were held consistent across both conditions of the study and the generalization probes. At the beginning of each session, the participant prompted the learner to sit at the table and the participant sat across or beside the learner. The participant placed five items in a line on the table about five inches apart within reach of the learner. The participant instructed the learner to “pick one.” The learner was given ten seconds to choose a stimulus. If the learner selected an item, he/she was given thirty seconds of access to the item (or able to consume the item, if edible). The participant recorded the learner’s response on the data sheet or on the tablet-based device. After thirty seconds, the item was removed from the immediate area. The remaining stimuli were rearranged by taking the item at the left end of the line and moving it to the right end and shifting the other stimuli, so they were equally spaced in an array in front of the learner. The participant again instructed the learner to “pick one.” The learner was given ten seconds to choose an item and given thirty seconds of access to the item (or until consumed). Once again, the response was recorded, the item removed, and the remaining items rotated. The assessment continued in this manner until all items were selected or the learner made no selection within ten seconds. If no selection was made, all remaining stimuli were removed, and the participant recorded “no selection” for those trials. If the learner selected more than one item, the learner was allowed to access the first item contacted. If two or more items were selected simultaneously, the learner was not allowed to access either item and the trial was reinitiated with the items rotated.

This procedure was repeated twice more so that the learner had access to all five stimuli in the array for a total of three times. Following the completion of the assessment, the participant finished filling out and scoring the data sheet (pen and paper condition) or reviewed the graphs provided by GAINS (AI condition).

#### Pen and Paper Condition

During all sessions (except generalization probes) in the pen and paper condition, the learner was an adult simulated learner who followed a randomly preselected script. Adult participants were given written instructions of the MSWO procedures (see Appendix A) as a job aid. The participants were given as much time as they needed to read the instructions and told to let the learner know when they were ready to begin. At this time, the stopwatch for the session was started and duration recording began. They were allowed to keep and refer to the written instructions throughout the session. The participants were told to conduct the assessment to the best of their ability but were not given any further instructions or feedback. If they asked questions, the primary interventionist would respond that they were to do whatever they thought was best. Adult participants were given the paper data sheet to fill out and record the learner’s selections. The stopwatch was kept running until the participant had completed and scored the assessment, generating a hierarchy of preferences.

#### AI Condition

**Fig. 1:**
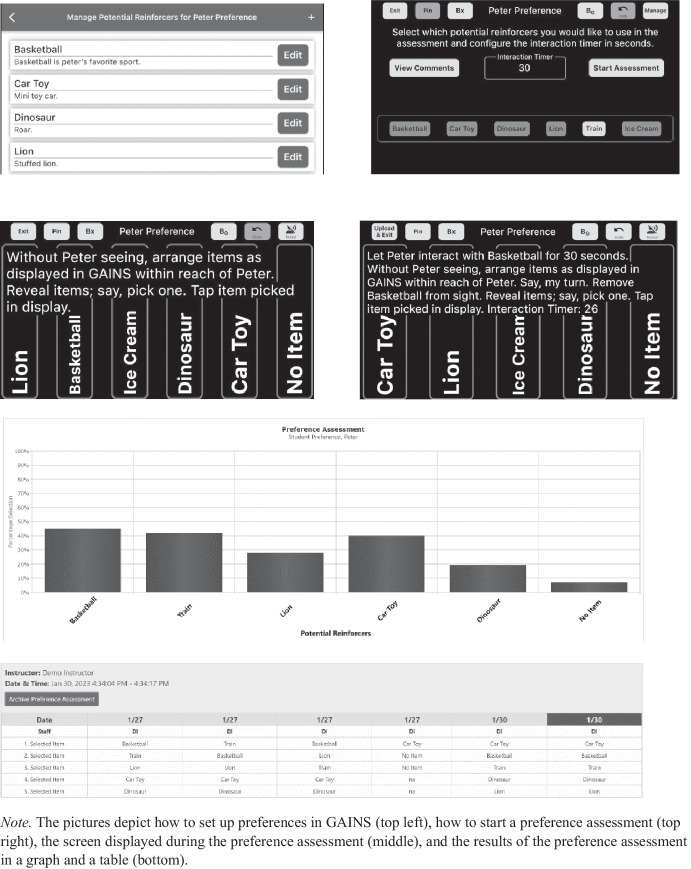
Screenshots of GAINS Application. Note. The pictures depict how to set up preferences in GAINS (top left), how to start a preference assessment (top right), the screen displayed during the preference assessment (middle), and the results of the preference assessment in a graph and a table (bottom)

During all sessions (except generalization probes) in the AI condition, the learner was an adult simulated learner. The simulated learner was told which script (selected at random) to follow prior to beginning each session. The participant used the AI application on a tablet device with a Bluetooth headset. The application provided guidance on conducting the MSWO via audio instructions on the Bluetooth headset. It also provided visual guidance using words and diagrams on the screen of the tablet device. Figure 1 shows example pictures of the display throughout the assessment. Prior to the first session using GAINS, each participant was given about five minutes to access the application on the tablet and complete a sample program not related to the study. The sample program was a task analysis for teaching a child handwashing. This program was used to familiarize the participants with how to log in to the application, interact with the icons, access the audio output with the Bluetooth headphones, and navigate within the application. The primary interventionist did not provide any guidance, feedback or answers to questions before, during, or after each trial or session. Every session was recorded. Following completion of the MSWO, the participant viewed the scores provided by GAINS and the primary interventionist asked the participant which item they would select to use in their program for the day to determine the participant’s interpretation of the results. In addition, as soon as the learner sat down at the table to begin the assessment, a stopwatch was started and kept running until the participant had viewed the results and generated a hierarchy of preferences among the stimuli selected.

#### Generalization Probes

Generalization probes occurred for both the pen and paper condition and the AI condition at the beginning and the end of the study. Generalization probes were conducted following the same MSWO procedures, but the learner was one of the child participants instead of the simulated learner. Stimuli for the child participants were rotated between sessions to prevent satiation effects.

#### Social Validity Survey

When all adult participants completed the final generalization probe, they were given a copy of the System Usability Scale survey (see Appendix C). They were asked to answer each question honestly and to the best of their ability. The social validity survey for the child participants was administered orally following completion of each day they were involved in the study.

### Data Analysis

Data collected on the primary dependent variable (fidelity of implementation) were displayed in graphical format and analyzed with visual analysis (Ledford & Gast, 2018). Descriptions of level, trend, variability, consistency, effect size, and immediacy were assessed. Effect size was calculated using nonoverlap of all pairs (NAP; Parker et al., 2011), a measure of effect size using nonoverlapping data. A NAP score of 0.00–0.67 is considered small, a score of 0.68–0.95 is considered moderate, and a score of 0.96 or above is considered large (Parker et al., 2011).

The secondary dependent measure of duration was displayed in a graphical format and the average duration for each condition with each participant was calculated. Data collected on the secondary dependent variable were also analyzed with visual analysis (Ledford & Gast, 2018) including descriptions of level, trend, variability, consistency, and immediacy.

The follow-up survey given to adult participants allowed respondents to choose from the following answer choices: strongly disagree, disagree, neutral, agree, and strongly agree. After completion of the survey, each response was assigned a numerical value ranging from 1 to 5 with 1 being “strongly disagree” and 5 being “strongly agree.” Data were analyzed by compiling responses and calculating results using an average and range of responses for each question. There was a total of 33 items on the follow-up survey relating to the treatment acceptability of GAINS and whether or not GAINS produced socially significant outcomes.

The social validity questionnaire administered to child participants allowed them to respond verbally and/or by pointing to a visual of a green smiley face, yellow face with a straight mouth, and red frowning face. Results from these questions were analyzed by calculating the percentage of responses in each category (i.e., smiley face, straight face, and frowning face) for each question.

## Results

**Fig. 2:**
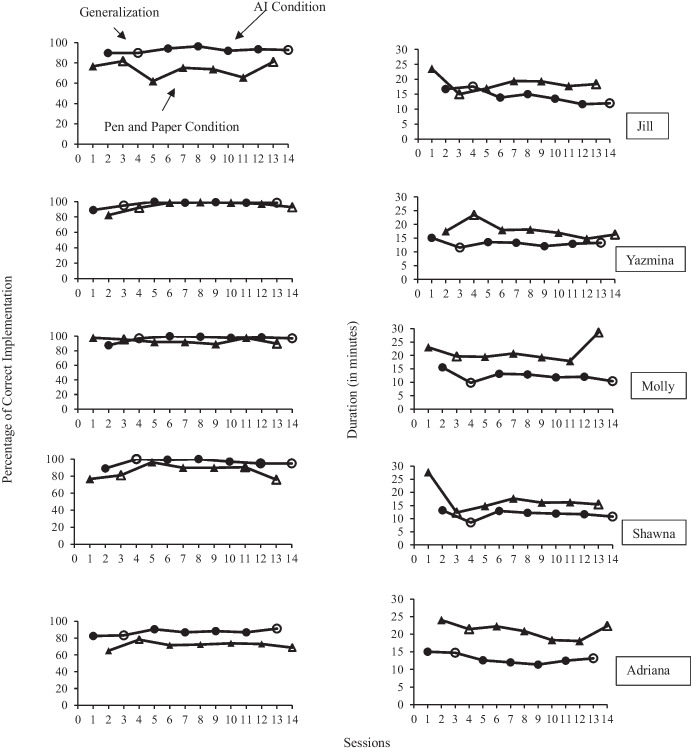
Implementation fidelity and duration graphs

Results for the primary measure of implementation fidelity and secondary measure of duration of assessment are represented graphically in Fig. 2. In addition, Table 2 shows the average, range and scoring errors for each participant under each condition, including generalization. Table 3 represents the average and range of duration for each participant under each condition. Overall, participants averaged 93.97% (range 82.48%-100%) during the AI condition compared to an average of 83.79% (range 62.04%-98.54%) during the pen and paper condition. NAP scores indicate that the difference had a moderate to large statistical effect for four of the five participants. In addition, the participants scored the results of the MSWO incorrectly in 16 out of 35 (45.71%) sessions during the pen and paper condition, including one participant who was unable to correctly score the MSWO during any of the pen and paper sessions. In contrast, all participants were able to accurately score and interpret the MSWO results during every session in the AI condition. On average, the MSWO was completed in 12 min 52.7 s (range 8 min 32 s-17 min 36 s) during the AI condition and 19 min 13.2 s (range 12 min 19 s-28 min 32 s) during the pen and paper condition. NAP scores indicate a moderate to large effect size for all five participants. Results of the follow-up survey suggest that adult participants found that AI had a high treatment acceptability and was capable of producing socially significant outcomes. Similarly, the child participants reported a high degree of treatment acceptability for both AI and pen and paper assessments, however they showed a stronger preference for AI.

**Table 2:** Implementation fidelity and scoring errors by participant and condition

Name	Standard Sessions				Generalization Sessions			
	Average (range) AI	Average (range) Pen and Paper	Scoring Errors AI	Scoring Errors Pen and Paper	Average (range) AI	Average (range) Pen and Paper	Scoring Errors AI	Scoring Errors Pen and Paper
Jill	92.60% (89.78- 96.35%)	73.72% (62.04- 81.75%)	0 (0%)	3 (60.00%)	91.24% (89.78, 92.70%)	81.39% (81.75, 81.02%)	0 (0%)	0 (0%)
Yazmina	96.98% (89.05–100%)	94.36% (82.48–99.2%)	0 (0%)	1 (20.00%)	96.72% (94.89, 98.54%)	92.34% (91.97, 92.7%)	0 (0%)	1 (50.00%)
Molly	96.45% (89.05- 100%)	85.71% (75.91- 96.35%)	0 (0%)	0 (0%)	97.45% (94.89, 100%)	78.47% (81.02, 75.91%)	0 (0%)	1 (50.00%)
Shawna	96.77% (87.59- 100%)	93.41% (89.78- 97.81%)	0 (0%)	3 (60.00%)	97.08% (97.08%)	92.70% (95.62, 89.78%)	0 (0%)	0 (0%)
Adriana	87.06% (82.48- 91.24%)	71.74% (64.96- 78.1%)	0 (0%)	5 (100%)	87.23% (83.21, 91.24%)	73.36% (68.61, 78.10%)	0 (0%)	2 (100%)
Total	93.97% (82.48–100%)	83.65% (68.61–95.62%)	0 (0%)	12 (48.00%)	93.94% (83.21- 100%)	83.65% (68.61- 95.62%)	0 (0%)	4 (40.00%)

**Table 3:** Duration of assessment by participant and condition

Name	Standard Sessions		Generalization Sessions	
	Average; range (in minutes: seconds) AI	Average; range (in minutes: seconds) Pen and Paper	Average; range (in minutes: seconds) AI	Average; range (in minutes: seconds) Pen and Paper
Jill	14:20; 11:40 -17:36	18:37; 15:01 – 23:33	14: 48; 17:36, 11:59	16:42; 15:01, 18:23
Yazmina	13:09; 11:35- 15:08	17:53; 14:48 – 23:28	12:27; 11:35, 13:19	19:39; 23:28, 16:19
Molly	12:15; 9:49 – 15:34	21:13; 17:54 – 28:32	10:07; 9:49, 10:25	24:06; 19:39, 28:32
Shawna	11:36; 8:32 – 13:10	17:03; 12:19 – 27:42	9:39; 8:32, 10:46	13:53; 12:19, 15:26
Adriana	13:03; 11:22 – 15:01	21:03; 18:04 – 24:01	13:57; 14:44, 13:09	21:54; 21:27, 22:20
Total	12:53; 8:32 – 17:36	19:13; 12:19 – 28:32	12:12; 8:32- 17:36	19:13; 12:19 – 28:32

### Jill

On average, Jill scored 18.88% higher on implementation fidelity during the AI condition than pen and paper. Using visual analysis, Jill’s data for the AI condition illustrate a high consistent level with little variability. Jill’s data for the pen and paper condition show a moderately high level with some variability. NAP for Jill’s fidelity of implementation was 1.00, indicating a large effect size with no overlapping data points. Jill made errors in scoring during three out of seven (42.86%) sessions during the pen and paper condition and did not make any scoring errors during the AI condition. In both conditions, Jill’s most frequent error was not providing sufficient time for the learner to access the selected item. However, she made almost three times as many errors in the pen and paper condition and had significantly more errors in failing to rotate the items accurately between trials.

In terms of duration of assessment, Jill averaged four minutes and seventeen seconds faster while using AI. Visual analysis for Jill’s data illustrates a positive low level with a decreasing trend and little variability in the AI condition. Whereas her data in the pen and paper condition demonstrate a moderate level with a slight decreasing trend and some variability. The NAP for Jill’s duration was 0.92, indicating a moderate effect size.

### Yazmina

Yazmina increased her implementation fidelity on average 2.62% while using AI. In terms of visual analysis, Yazmina’s data while using AI demonstrate a consistent high level with no variability. Yazmina’s data in the pen and paper condition illustrate a high level with little variability. NAP for Yazmina’s implementation fidelity was 0.67, indicating a small effect size. Yazmina made errors in scoring two out of seven (28.57%) sessions during the pen and paper condition and did not make any scoring errors during the AI condition. Yazmin’s most frequent error during the AI condition was not rotating the items between trials and this happened most during her first two sessions. During the pen and paper condition, her most frequent error was not providing sufficient time for the learner to access the chosen item.

Yazmina completed the MSWO four minutes and forty-four seconds faster on average during the AI condition. Using visual analysis, Yazmina’s data under the AI condition show a positive stable low level. In contrast, her data during the pen and paper condition demonstrate a stable moderate level. NAP for Yazmina’s duration was 0.98, indicating a large effect size.

### Molly

Molly scored on average 10.74% higher on implementation fidelity during the AI condition in comparison to the pen and paper condition. Molly’s data demonstrate a high level with no variability under the AI condition. In contrast, Molly’s data under the pen and paper condition indicate a moderately high level with a small degree of variability. Her implementation fidelity data obtained a NAP score of 0.88, indicating a moderate effect size. Molly made errors when scoring the assessment in one out of seven (14.29%) sessions during a generalization session in the pen and paper condition. She did not make any scoring errors during the AI condition. During the AI condition, Molly’s most common error was in failing to space the items appropriately in front of the learner, whereas in the pen and paper condition, her most common error was in failing to provide enough time for the learner to contact the selected items.

On average, Molly completed the MSWO almost eight minutes faster using AI. In terms of visual analysis, Molly’s data under the AI condition demonstrate a positive low level with a slight degree of variability. Her data under the pen and paper condition illustrate a moderate level with an increasing trend and some variability. NAP for Molly’s duration was 1.00, which indicates a large effect size with no overlapping data points.

### Shawna

Shawna averaged 3.36% higher in implementation fidelity while using AI. In terms of visual analysis, Shawna’s data illustrate a high level with no variability in the AI condition. Shawna’s data during the pen and paper condition demonstrate a high level with some variability. NAP for Shawna’s fidelity of implementation was 0.77, indicating a moderate effect size. Shawna made errors when scoring the assessment in three out of seven (42.86%) sessions during the pen and paper condition and zero out of seven (0%) sessions during the AI condition. Shawna’s most frequent error in the AI condition was failing to space the items appropriately in front of the learner. Her most frequent error in the pen and paper condition was failing to provide adequate time for the learner to access the selected item.

Shawna completed the MSWO on average five minutes and thirty-five seconds faster using AI. Using visual analysis, her data in the AI condition demonstrate a positive low level with some variability. Her data during the pen and paper condition illustrate a moderate level with a decreasing trend and a high degree of variability. NAP for duration for Shawna was 0.96, indicating a large effect size.

### Adriana

Adriana scored on average 15.32% higher on implementation fidelity while using AI. In terms of visual analysis, Adriana’s data for AI show a high level with an increasing trend and no variability. In contrast, Adriana’s data during the pen and paper condition illustrate a moderately stable high level. Adriana’s data for implementation fidelity obtained a NAP score of 1.00, which indicates a large effect size with no overlapping data points. Adriana made scoring errors during all seven sessions (100%) in the pen and paper condition. In contrast, she made no errors in scoring during the AI condition. During the AI condition, Adriana’s most frequent error was in failing to space the items appropriately in front of the learner. During the pen and paper condition, Adriana made approximately the same number of errors in spacing, but her errors in failing to allow the learner adequate time with the chosen item and failing to rotate the items between trials increased significantly.

Adriana completed the MSWO an average of eight minutes faster using AI. For visual analysis, Adriana’s duration data in the AI condition demonstrate a positive stable low level. Her data under the pen and paper condition illustrate a stable moderate level. NAP for Adriana’s duration was 1.00, indicating a large effect size with no overlapping data points.

### Adult Participant Social Validity Survey

There was a total of 33 items on the social validity survey. The questions related to the treatment acceptability and social significance of using GAINS for preference assessments compared to pen and paper self-instruction. Table 4 reflects a summary of the most relevant items and general results. Overall, the responses averaged 4.44 (range 1–5). The lowest scoring items involved the use of the audio assistance on GAINS and included, “Audio is enough. It is not necessary to read the display” and “Audio assistance is useful,” which scored an average of 2.6 (range 1 -5) and 3.8 (range 3–5), respectively. All other items scored an average between 4.0 and 5.0. The highest scoring items involved the ease of interaction, including the ability to learn to use it and the ease of reading the display screen. Only one participant added a write-in comment, which read “I think that the app was far easier to use, and you do not have to do math!”.

**Table 4:** Social validity- acceptability

Question	Mean and Range
Learning to use GAINS is easy for me	5
Interaction with GAINS is clear and easy to understand for me	4.8 (range 4–5)
If I have a choice, I would use GAINS in helping consumers learn new skills	4.5 (range 4–5)
I can use GAINS even if there is no one to help me	5
It is easy to login into and start instruction with GAINS	4.8 (range 3–5)
I can hear the audio assistance provided	4.8 (range 4–5)
It is easy to know what to do next	5
Audio assistance is easy to follow and useful	4 (range 3–5)
The display is easy to read	5
Audio is enough. It is not necessary to read the display	2.6 (range 1–5)
It is easier than pen and paper to input data	4.4 (range 2–5)
Data recording is more accurate than pen and paper	4.6 (range 3–5)
It is useful to be provided error correction for a step and easy to tell what the error correction is for a step	4.4 (range 3–5)
It is useful that GAINS tracks preference of each item	5
The use of GAINS in helping consumers learn new skills enhances my productivity	4.6 (range 4–5)
Generally, I consider GAINS can be useful to me in helping consumers learn new skills	4.6 (range 4–5)
GAINS makes it easy to track preference of each item	4.8 (range 4–5)
GAINS improves Preference Assessment administration	5
It is important for consumers to identify preferences	5
Using Preference Assessment to identify preferences is useful	5
Learning how to conduct Preference Assessments is useful	5

### Child Participant Social Validity Survey

The follow-up social validity questionnaire for the child participants contained five items. Although there were only two child participants, they completed the social validity questionnaire after every day they were involved with the study, resulting in two different survey responses for each child and a total of four responses, represented in Table 5. The children indicated that they enjoyed having choices all the time. The children indicated a slightly higher treatment acceptability for the tablet over the pen and paper. In general, th.

**Table 5:** Social validity responses for child participants

Question	Percentage Responding “Yes” or Green Smiley Face	Percentage Responding “I Don’t Care” or Yellow Face	Percentage Responding “No” or Red Frowning Face
Did you like having choices of things to (play with) or (to eat)?	100%	0%	0%
Did you like it when the teacher used the paper?	75%	0%	25%
Did you like it when the teacher used the tablet?	75%	25%	0%
Did you have fun today?	100%	0%	0%
Question	Percentage Responding “Tablet”	Percentage Responding “It Doesn’t Matter”	Percentage Responding “Paper”
Which one did you like more the paper or the tablet or it doesn’t matter?	75%	25%	0%

## Discussion

The purpose of the current study was to compare the use of typical pen and paper self-instruction to AI during preservice SLPs’ training and implementation of MSWO. Fidelity of implementation, duration, and social validity were evaluated under both conditions. Results indicated that AI reduced the duration of assessment by seven minutes (32.84%) on average and produced equal or better implementation fidelity for all five participants. In addition, all adult participants indicated that AI was easier and more enjoyable to use. Child participants showed a preference for AI over pen and paper and indicated that AI had a high treatment acceptability.

These results are consistent with previous research which suggests that pen and paper self-instruction is insufficient to produce consistent outcomes during SPA training and implementation (Graff & Karsten, 2012b). Previous research has attempted to remedy this by providing a written job aid that can be referenced during implementation. However, this was still insufficient for some professionals to implement SPA with fidelity, necessitating additional time and resources spent on training (Graff & Karsten, 2012b; Shapiro et al., 2016). Research using technologies, such as telehealth and computer-aided instruction, have improved outcomes, yet even successful interventions have not overcome the limitations of lengthy initial training phases and the requirement of expert trainers (Hansard & Kazemi, 2018; Lipschultz et al., 2015; Rosales et al., 2015; Weston et al., 2020; Wishnowski et al., 2017). The results of the current study demonstrate that AI, such as the GAINS application, may provide a solution that overcomes these limitations and can train professionals to achieve high levels of fidelity during SPA implementation.

Both Jill and Adriana had a NAP score of 1.00 for implementation fidelity, which not only indicates a large effect size, but shows that their data contain no overlap. Their lowest score in the AI condition was higher than their highest score in the pen and paper condition. Although the other participants showed increased scores in the AI condition, the differences may be harder to assess due to the ceiling effect of having high levels of responding across both conditions. Because four out of five participants initially scored higher using the GAINS application, it could indicate that AI may be better able to produce high levels of fidelity for most professionals without extensive training or practice.

One important finding is that the high levels of implementation fidelity observed during standard sessions were immediately transferred to generalization sessions with children for all five participants. There was no need for additional training or feedback from an expert. This could be especially valuable in a clinical setting, where experts have extensive time demands. Perhaps the most significant finding of this study is the difference in scoring and interpretation errors between the two conditions. In total, participants made scoring errors in 16 out of 35 (45.71%) sessions during the pen and paper condition. Whereas no errors in interpretation were made while using AI. Moreover, one participant (i.e. Adriana) was never able to correctly score any of the pen and paper condition sessions. In contrast, after viewing the results provided by GAINS, she was able to interpret them correctly during every session. Even participants with high levels of fidelity, such as Yazmina, Molly, and Shawna, scored the MSWO incorrectly on at least one occasion. Incorrect scoring of the MSWO produces an inaccurate preference hierarchy, which in a clinical setting could lead to unproductive treatment programs and erroneous decisions about which stimuli should be included in instructional programming. In addition, the one written comment on the follow-up survey stated that GAINS was “far easier to use, and you do not have to do math!”, which suggests the participants may have found the scoring of the pen and paper assessments particularly challenging and unenjoyable.

Another significant finding is that participants performed the MSWO on average seven minutes faster using AI. Both Molly and Adriana had a NAP score of 1.00, indicating there were no overlapping data points. Their longest session using AI was still shorter than their fastest session using pen and paper. In addition, all participants were initially able to perform the MSWO more quickly with AI, regardless of whether or not they had ever completed a pen and paper assessment. When combined with the results of the follow-up survey, in which participants consistently indicated that GAINS was easy to read and learn to use, it suggests that professionals could save a significant amount of time and energy by using AI for SPA.

### Clinical Implications

The use of preferred stimuli during educational programs can increase social engagement, enhance skill acquisition, promote prosocial behavior, and provide individuals with IDD more opportunities for autonomy and choice-making (Hu et al., 2021; Logan & Gast, 2001; Phillips et al., 2017; Rajaraman et al., 2022). Leveraging preferred stimuli during instructional programs could increase communication development and impact overall quality of life for individuals with IDD by supporting them in developing the skills needed to engage and participant meaningfully in their communities while simultaneously making instruction more enjoyable (Logan & Gast, 2001; Overmars-Marx et al., 2014). Research has demonstrated that without the use of SPA, professionals are often unable to identify preferred items accurately (Cote et al., 2007; King, 2016). Providing SLPs and other professionals with a fast and easy method of performing SPA could be considered an essential job function and foundational to the implementation of many evidence-based practices (Graff & Karsten, 2012a; Steinbrenner et al., 2020).

Previous training methods have relied heavily on the use of expert trainers, creating many obstacles for professionals that lack training and procedural knowledge. Many organizations in rural or impoverished areas may not have access to experts for effective initial training or follow-up sessions if needed. In this study, all five participants were able to complete the MSWO with fidelity at or above 80% upon initial use of AI with no training or feedback from experts. AI might eliminate the need for training by experts and provide a viable alternative in areas with limited access to experts.

In addition to decreasing the need for experts, using AI for conducting regular SPA could save professionals valuable instructional time. In the current study, AI saved participants an average of seven minutes per assessment. If a SPA is conducted every day, which would be recommended given the complexity of factors involved in motivations (Bishop & Kenzer, 2012), the use of AI would save a professional over 35 min per week, or over two hours per month. This represents a significant amount of time that could be utilized in teaching new skills, while still identifying the most effective reinforcers every day. Given that SLPs may only see a child for one to two hours per week, additional instructional time would be even more valuable (Gillon et al., 2017).

Participants in this study expressed a consistent preference for AI, specifically finding it more effective, easier to use, and more accurate than pen and paper assessments. If professionals had an effective, efficient, and preferred method of implementation, it might result in SPA being conducted more frequently, which could produce improved instructional outcomes for students and clients. Additionally, giving professionals the tools to perform their jobs more consistently, accurately, easily, and quickly could significantly reduce their stress levels, leading to higher job satisfaction and staff retention rates.

However, AI is not the only solution to the challenges professionals face in conducting preference assessments. For example, professionals might be able to use a simple, single-trial MSWO to identify a reinforcer for a session. Some organizations may find that occasionally monitoring staff fidelity is sufficient for maintaining treatment outcomes. The initial cost of AI might be prohibitive for smaller organizations. Individual clinicians will need to consider a myriad of factors in determining which solution will support their needs. Nevertheless, the results of this study support the use of AI as one potential solution.

#### Limitations

Due to the nature of the alternating treatment design, it is possible that there were carryover effects from one treatment condition to the other. Instructions provided in one condition could have affected performance and the way the instructions were interpreted or remembered in the other treatment condition. For example, participants may have become dependent on the instructions provided by the GAINS technology, which could have precluded higher scoring in the pen and paper condition. Perhaps if these two conditions had been run separately, they may have mastered the pen and paper condition more readily. However, four out of five participants had higher initial fidelity scores while using GAINS, which suggests that there may not have been significant carryover effects. In addition, the initial treatment condition varied across participants and there was a minimum of forty-eight hours between sessions to control for rapid alternation effects and sequencing effects.

Another limitation is that the initial time to set up a profile on GAINS was not included in the session duration. A student profile must be set up upon initial use of GAINS and includes entering information, such as first and last name and birthdate. The student is stored in GAINS and only needs to be selected for future sessions. In addition, to use the preference assessment feature, each item used must be entered separately into the program with an optional description. Each of these items is stored in the program and only needs to be selected for future sessions. Therefore, the initial session when both student data and item information must be entered took an additional few minutes, however during subsequent sessions, only the correct student and relevant items needed to be selected from the list on the program. Neither the initial set up nor individual session set up was included in the duration of assessment. In an attempt to remain consistent, the time for each participant to fill out the top of the pen and paper data sheet and record each item during the pen and paper condition was not included as well.

There were several limitations presented by the participants of the study. Firstly, adult participants represented a higher level of education than some professionals working with individuals with IDD. This may have contributed to higher levels of implementation fidelity in both conditions. Secondly, the child participants both presented with vocal capabilities and typical development. Therefore, results may not generalize to child populations with IDD or limited communication abilities. 

Finally, the primary and secondary observer could not be blind to treatment conditions because it was clear on the video recording if the participant was using a tablet or pen and paper. In order to eliminate as much observer bias as possible, the secondary observer who completed the IOA did not learn about the application or any other studies that have been completed with the application. However, the primary observer was very familiar with the application and had previously conducted a study using a different program of the application (Griffen et al., 2023).

#### Future Research Directions

Future research should extend these findings by using a different population of professionals or caregivers as implementors of SPA. Since caregiver report of reinforcers is characteristically unreliable (Brodhead et al., 2017; Cannella-Malone et al., 2005, 2013; Graff & Karsten, 2012a), AI may allow caregivers to identify more effective reinforcers and preferred stimuli. As stated in the limitations, this study included participants with a fairly high level of education and training that some professionals working with children with IDD might not have. Future research should evaluate how a professional’s level of education affects their ability to implement SPA accurately using AI.

Additionally, the participants in the study were all female between the ages of twenty and twenty-two, which represents a very restricted population. Future research should include older participants who may have less experience with technology and may face additional barriers in its use. Inclusion of male participants would also be valuable. As stated in the limitations, the child participants were not individuals with IDD or limited communication abilities. Due to the primary investigator’s limited access to child participants, MSWO sessions were conducted outside of typical learning programs or educational sessions. Future research should conduct sessions as a part of typical school days or therapy sessions.

The current study chose to use written instructions of the procedures as a comparison condition based on its use in previous research (Hansard & Kazemi, 2018; Higgins et al., 2017; Rosales et al., 2015; Roscoe & Fisher, 2008; Roscoe et al., 2006) as a baseline or standard of practice. However, future research should compare GAINS to other technology-based training methods, such as computer-aided self-instruction, video modeling, and telehealth with multimedia presentations. These further evaluations may present a more detailed analysis of what, if any, advantages users can expect from GAINS. The current study did not include a detailed analysis of time-saving qualities (i.e. time during assessment, scoring, preparation, etc.) or of the exact nature of participant errors across conditions. Future research including such analyses would be valuable in producing more accurate technology and resources for practitioners.

Lastly, MSWO was the only type of SPA used in this study. Research has shown that some children who have significant behavioral challenges or have not yet developed scanning abilities may benefit from other forms of SPA (Tung et al., 2017). Therefore, research should examine if AI can be used to achieve high levels of implementation fidelity with other forms of SPA, such as paired stimulus, multiple stimulus with replacement, and free operant. This would hold high practical value for professionals who may need to conduct a variety of SPA in clinical or school settings.

## Conclusion

Results demonstrated that AI produced high levels of implementation fidelity with shorter assessment durations for five preservice SLPs, when compared to traditional pen and paper assessments. Pen and paper assessments resulted in a significant number of scoring errors and produced inaccurate preference hierarchies almost half the time. In contrast, AI produced an accurate preference hierarchy in every session that was easily interpreted by the participants. Moreover, AI was viewed as having a higher treatment acceptability and producing more socially significant outcomes by all five adult participants. Child participants reported a higher degree of treatment acceptability for AI, as well.

Since professionals have cited a lack of procedural knowledge and lack of time as the greatest barriers to regular SPA implementation, AI may provide a solution that would allow professionals to complete them more easily, accurately, and efficiently. This might lead professionals, such as SLPs, to conduct SPA more regularly, allowing individuals with IDD to express their preferences and creating opportunities to support client control, choice-making, and autonomy while facilitating engagement and skill development that could lead to more meaningful social interaction and inclusion in their communities. As such, utilizing AI to teach professionals could reduce communication and language knowledge and skill barriers for individuals with IDD, and in turn increase social inclusion, by reducing the knowledge and skill barriers of the professionals who support them.

## Data Availability

The data that support the findings of this study are available from the first author upon reasonable request.

## Appendix A: Pen and Paper Written Instructions

Multiple Stimulus Without Replacement (MSWO; adapted from DeLeon & Iwata, 1996).

### Procedure

Prior to each session, participants were instructed or prompted to sit in one of the chairs; the experimenter sat in the other. Five items per participant were chosen for presentation during each assessment. A selection response was recorded when the participant made physical contact with one of the presented items. The experimenter also served as an observer and recorded selections on data sheets that were customized for the MSWO procedure. The primary dependent variable consisted of a percentage score indicating the number of times an item was selected over the number of trials during which the item was presented. This score was then used to rank the items from most-to-least preferred.

Prior to the beginning of the first session, participants were given 30-s access to each of the leisure items. Subsequently, participants were exposed to one or two assessment sessions per day. Each session began with all items sequenced randomly in a straight line on the table about 5 inches apart. While a participant was seated at the table approximately ½ -to- 1 ft. from the stimulus array, the experimenter instructed the participant to “pick one.” The participant had 10 s to select an item. If the participant selected an item, the participant received 30-s access to the item. After the participant received access to the item, the item was removed from the immediate area. Prior to the next trial, the sequencing of the remaining items was rotated by taking the item at the left end of the line and moving it to the right end, then shifting the other items so that they were again equally spaced on the table. The second trial then followed immediately. This procedure continued until all items were selected or until a participant made no selection within 10 s from the beginning of a trial. In the latter case, the session ended (all remaining stimuli were removed), remaining stimuli were recorded as “not selected,” and a new session was initiated. If the participant made contact with more than one item, the participant was allowed access to the first item contacted, and that item was recorded as the selection. If two or more items were simultaneously selected, participants were not allowed access to the items and the trial was reinitiated (i.e., the stimuli were rotated and they were again asked to “pick one”). If the participant simultaneously selected two items a second time, the session ended. If the participant grabbed an item that was not in the array, the grabbed item was removed, and the therapist continued with the current trial.

## Appendix C: Usability Survey for Adult Participants

Thank You!
